# Quiescence of *Escherichia coli* Aerosols to Survive Mechanical Stress during High-Velocity Collection

**DOI:** 10.3390/microorganisms11030647

**Published:** 2023-03-03

**Authors:** Brooke L. Smith, Maria D. King

**Affiliations:** Aerosol Technology Laboratory, Biological & Agricultural Engineering Department, Texas A&M University, College Station, TX 77843, USA

**Keywords:** mechanical stress, low cutpoint wetted wall cyclone, pressure drop, quiescence, dormancy, antibiotic resistance

## Abstract

A low cutpoint wetted wall bioaerosol sampling cyclone (LCP-WWC), with an aerosol sampling flow rate of 300 L/min at 55″ H_2_O pressure drop and a continuous liquid outflow rate of about 0.2 mL/min, was developed by upgrading an existing system. The laboratory strain *Escherichia coli* MG1655 was aerosolized using a six-jet Collison Nebulizer and collected at high velocity using the LCP-WWC for 10 min with different collection liquids. Each sample was quantitated during a 15-day archiving period after aerosolization for culturable counts (CFUs) and gene copy numbers (GCNs) using microbial plating and whole-cell quantitative polymerase chain (qPCR) reaction. The samples were analyzed for protein composition and antimicrobial resistance using protein gel electrophoresis and disc diffusion susceptibility testing. Aerosolization and collection were followed by an initial period of quiescence or dormancy. After 2 days of archiving at 4 °C and RT, the bacteria exhibited increased culturability and antibiotic resistance (ABR), especially to cell wall inhibitors (ampicillin and cephalothin). The number of resistant bacteria on Day 2 increased nearly four-times compared to the number of cells at the initial time of collection. The mechanical stress of aerosolization and high-velocity sampling likely stunned the cells triggering a response of dormancy, though with continued synthesis of vital proteins for survival. This study shows that an increase in intensity in environmental conditions surrounding airborne bacteria affects their ability to grow and their potential to develop antimicrobial resistance.

## 1. Introduction

Research has shown that environmental effects, such as temperature change or osmotic stress, can cause a physical and molecular response from bacteria, including quiescence [1]. Quiescence is a state in which cells are not growing or replicating but are able to return to replication, and growth showed that Gram-negative bacteria respond to mechanical (tensile, shear and compressive) stressors using molecular mechanisms [2,3].

Turgor pressure in stable *E. coli* is ~2–4 atm but *E. coli* can survive with transient changes when turgor pressure changes to 10–20 atm. The protective outer membrane and cell wall are the first to experience stress and the first line of defense to protect the cell when it experiences unfavorable conditions. A change involving osmotic downshock can trigger pressure changes of 10 atm inside the cell in a matter of seconds. To avoid cell lysis, cells can gate or open their mechanosensitive and other membrane channels to alleviate pressure [4]. It is possible that some of these major changes can cause a change in cell mechanics [5].

In the event of hyperosmotic stressors or environmental changes surrounding the bacterium, *E. coli* cells can activate antimicrobial responses, including dormancy and growth arrest. The activation of dormancy does not indicate that the cell’s functioning stops completely; it only indicates that it has limited functioning to conserve energy and continue to survive in stressful situations. The regulation of genes and proteins can increase or decrease depending on whether the cells need increasing ATP synthases, histidine or other proteins for survival. While upregulation of some genes may occur to support the cells’ vital processes, downregulation can also occur if expression of certain genes is not necessary and if it helps conserve energy [6,7]. For example, *E. coli* expresses a stress response regulator RpoS, which allows the bacterium to regulate its growth for survival. In preparation for instant and prolonged stress, the cells can begin a slow growth overseen by RpoS. Patange et al., 2018 imaged the growth feedback model when RpoS is regulating growth to show the slowing of single-cell growth in its absence or presence [8].

Protein synthesis is inhibited when cells enter quiescence [9]. Protein synthesis is one of the highest-energy and costliest processes of a cell [10]. When a cell encounters a stressful environment, protein synthesis does not cease and ribosomal proteins stay active. Bacteria can downregulate ribosomal activity to decrease the amount of proteins synthesized in stressful environments and quiescence. Franken et al., 2017 found that it is costly to remove or rid the cell of ribosomes, but bacteria are able to downregulate the activity to decrease synthesis of other proteins in stressful environments [11].

Studies have also shown that environmental changes and stressors can induce an antibiotic resistance response. Zhou et al., 2015 highlighted three tiers of defense mechanisms for bacteria, starting with biofilms, then outer membrane functions, and then intracellular defenses. For clustered or singular aerosolized bacteria, they must use the second line of defense in a disrupted microbiome, which is their cell wall, outer membrane, and efflux pumps [12]. They act as a barrier of antimicrobials, and efflux pumps can act as a gateway to rid the cell of unwanted outer substances that penetrated the cell [12]. Recent research demonstrates that aerosolization triggers antibiotic resistance within minutes in *E. coli* [13].

To aerosolize the bacteria for collections, the six-jet Collison nebulizer was used with 20 psi of pressured air, allowing a liquid suspension of bacteria to be broken into smaller droplets that impact the inner walls of the glass jar portion of the nebulizer, creating an aerosol. Nebulization was limited to ten-minute periods as the shear forces involved in the atomization can cause physiological damage to the individual cells [14,15]. Munita and Arias, 2016 designed a study to use a high-velocity sampler to improve the collection efficiency of bioaerosols and to analyze the effect of environmental changes in the functions of *E. coli* cells [16]. The wetted wall cyclone (WWC) collectors used in this study collect and concentrate bioaerosol particles in a liquid [17]. The WWC is equipped with an airblast atomizer to create a liquid spray that coats the inner wall of the cyclone with a liquid film, with thickness in the order of tens of micrometers. Aerosol particles entering the WWC inlet are primarily deposited by the mechanism of inertial impaction onto the liquid film that serves as an effective collector for the aerosol particles when it breaks down into a small stream of liquid to be aspired by an external pump [18]. The advantage of the WWC being a near-continuous liquid flow system is that high-time-resolution samples are provided with a small consumption of collection liquid and the culturability and DNA integrity of the bioaerosols is maintained during collection [17,19]. The Low Cutpoint Wetted Wall Cyclone Bioaerosol collector used in this study has an average aerosol-to-hydrosol collection efficiency of 98%, with a cutpoint (particle size where the collection efficiency is 50%) of <0.3 μm aerodynamic diameter (AD), operating at 300 L/min with 98 m/s velocity [20].

The change in antibiotic resistance, culturability and other behaviors in bacteria are important to study under environmental changes, especially in high-risk facilities, such as hospitals and meat production facilities, that already have a heightened amount of bioaerosols. Smith and King 2022 showed that the exposure of *E. coli* cells to the stress of aerosolization resulted in more antibiotic resistance, an increase in gene expression of antibiotic resistance genes (ARGs), specifically an increased resistance to cephalothin, gentamycin and tetracycline antibiotics and an increase in expression of efflux pumps [13]. One commonality between high-risk indoor facilities is the need for a high-functioning heating ventilation and air conditioning (HVAC) system. The addition of an HVAC system offers benefits, such as maintaining clean air and climate control [21]. Along with other benefits, naturally, the HVAC systems introduce mechanical stress on bioaerosols from pressure, velocity, and relative humidity (RH) of the air [22]. Hospitals must maintain a mandatory minimum of six air exchanges per hour (ACH), increased to 12–15 ACH in infectious units, which takes approximately 1 h or 28–35 min, respectively, to remove airborne contaminants at an efficiency of 99.9% [23]. The increased ACH creates an increased air velocity [23].

The goal of this study was to analyze the effect of high-velocity aerosolization and collection using a recently developed high air volume, high-velocity Low Cutpoint Wetted Wall Cyclone bioaerosol collector on the culturability, DNA integrity, protein synthesis and antibiotic resistance of fresh, mid-log phase Gram-negative bacteria. The study utilized the nonpathogenic strain *Escherichia coli* MG1655 (genotype *E. coli* K-12, λ-, F-, rph-1) derived from parent strain W1485 by acridine orange curing of the F plasmid. This well-studied bacterium was used as the simulant for aerosolized pathogenic bacteria collected with the Low Cutpoint wetted wall cyclone (LCP-WWC) highlighted in this study.

## 2. Materials and Methods

### 2.1. Bacterial Aerosolization and Collection

Mid-log phase fresh cultures of *E. coli* K-12 MG1655 (*E. coli* Genetic Resources at Yale CGSC, The Coli Genetic Stock Center, New Haven, NE, USA) were grown in Luria Bertani (LB) medium for about 3 h at 37 °C with constant shaking at 0.102 g [24]. The cells were harvested by pelleting them in a centrifuge at 2880× *g* for 7 min and re-suspending them in deionized milli-Q (MQ) water that contained 10% phosphate buffer saline with 0.1% Triton X-100^®^ surfactant (PBST) at a pH of 7.4. All the reagents and buffers used in the study were sterilized by autoclaving.

The cell suspensions, in 30 mL batches, were aerosolized with a 6-jet Collison atomizer (BGI, Waltham, MA, USA) operated at 140 kPa (20 psi), which provides an aerosol output of 12 L/min. Based on the concentration of *E. coli* in the suspension and the atomization rate, approximately 460,000 viable cells were generated during any 10 min test period [19].

Single vegetative *E. coli* aerosols were collected with the Low Cutpoint Wetted Wall Cyclone (LCP-WWC) at 300 L/min airflow (∆p = 55″ H_2_O) and room temperature (RT, 25 °C), transiently exposing the bacterial aerosols to 98 m/s air velocity for seconds during collection. The samples were collected in 0.01% Tween-20 for 10 min simultaneous aerosolization collection periods and divided into two aliquots. One aliquot was analyzed as collected; these samples were denoted as Tween. To the second aliquot, phosphate buffer saline (PBS) at pH 7.4 was added to 10% final concentration; these samples were denoted Tween + 10PBS. The third type of sample was collected in 0.01% Tween 20 for five minutes into tubes containing PBS to 10% final concentration; these samples were denoted 10PBS + Tween. Day 0 samples were all analyzed immediately after testing and, thus, did not have an archiving temperature.

In the test, aerosol output from the Collison generator was fed into 1″ diameter vinyl tubing connected directly from the nebulizer and directed into the inlet of the LCP-WWC. The generated bacterial particles were continuously sampled with the cyclone collector over a 10 min period, during which the specific collection liquid was pumped into the air blast atomizer of the LCP-WWC. The aerosol generation was then stopped but operation of the LCP-WWC continued for an additional 2 min to allow for flushing of residual particulate matter from the cyclone. At least triplicate tests were run with each bacterium suspension. A washing period of five minutes with MQ water was inserted between the sampling tests. At the end of the tests, the weight of each hydrosol sample was measured.

As a reference sampler, the SKC BioSampler (SKC Inc., Eighty Four, PA, USA) was used at 12.5 L/min airflow for 10 min collections of *E. coli* aerosols into 20 mL of 0.01% Tween-20. Each of the BioSampler’s three tangential nozzles acts as a sonic orifice, permitting 4.2 L/min airflow per nozzle [25]. Although the BioSampler is sampling at a significantly lower airflow rate (12.5 L/min) compared to the WWC collector in this study (300 L/min), it was selected as a reference sampler because it is widely used, commercially available and serves as a reference for bioaerosols being exposed to sonic speeds. The SKC BioSampler has a 79% collection efficiency for particles between 0.3 μm and 2 μm and a 100% collection efficiency for particles greater than 2 μm [26]. Culturability was not maintained after Day 1 of archiving at 4 °C and RT; therefore, only the SKC Day 0 values are included in this study. The collected samples and the stock suspensions from the LCP-WWC sampler were divided into two equal volumes and stored at room temperature (RT) and 4 °C, respectively, for a period of 15 days. During archiving, the culturability, antibiotic resistance and DNA intactness of the samples were monitored daily by plating and for the samples archived in 10% PBS, by quantitative real-time Polymerase Chain Reaction (qPCR). These methods are further explained in Section 2.2, Section 2.3, Section 2.4, Section 2.5, Section 2.6 and Section 2.7.

### 2.2. Aerosol Particle Sizing

**Figure 1:**
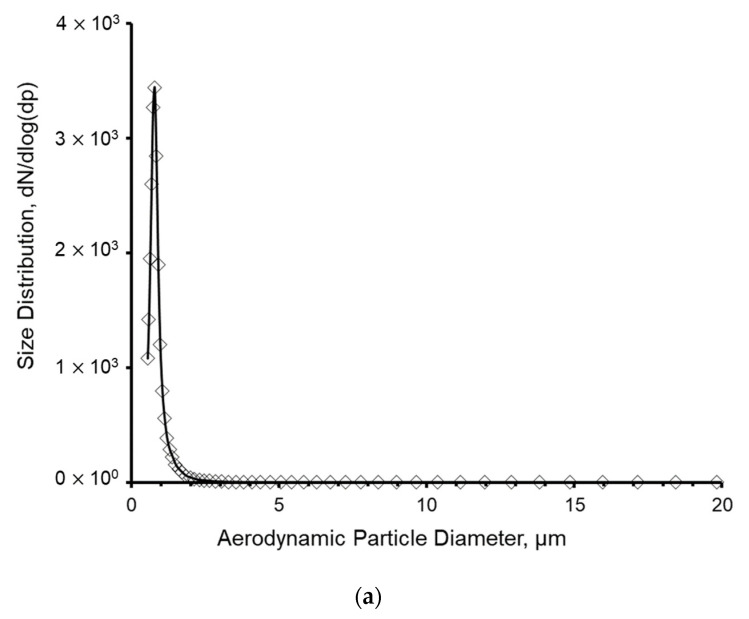

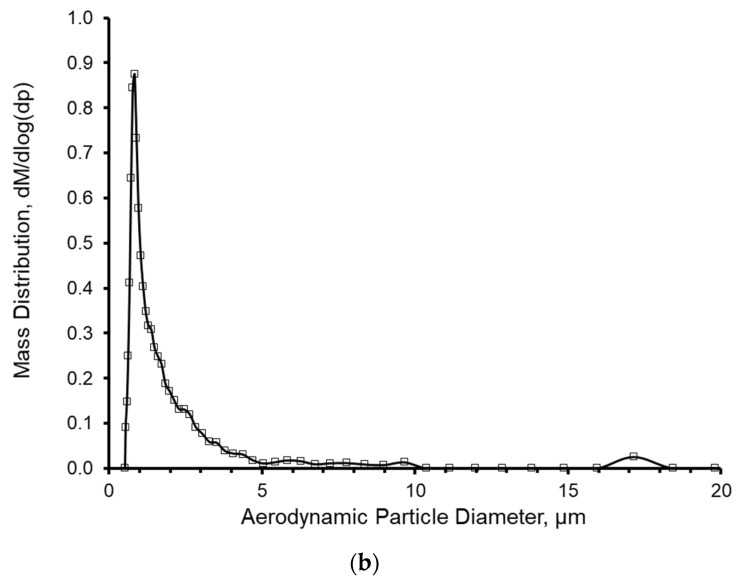
(**a**) The size distribution of droplets given by dN/dlog(dp). (**b**) The mass distribution given by dM/dlog(dp) during the aerosolization of the *E. coli* MG 1655 suspension.

The average size of the resulting aerosol particles, determined with an Aerodynamic Particle Sizer (APS, Model 3321, TSI Inc., Shoreview, MN, USA) upstream of the LCP-WWC sampler, where the 5 L/min aerosol flow rate for the APS was extracted from the flow duct, was approximately 1 µm aerodynamic diameter (AD) (Figure 1).

### 2.3. Plating and Analysis

Culturable colony forming units (CFUs) were determined by plating 100 µL of appropriate dilutions of the samples to result in 60–200 colonies on Difco Tryptic Soy Agar (TSA) plates (Becton, Dickinson and Co., Sparks, MD, USA). Colonies were counted after 18 h overnight incubation of the plates at 37 °C.

### 2.4. Quantitative Polymerase Chain Reaction (qPCR)

The whole-cell quantitative PCR (qPCR) reaction mixture (10 µL total) contained the DNA template (3 µL of the collected samples), the forward and reverse primers (16S 1369 forward ‘5-AAGTCGTAACAAGGT’ and 1492 reverse ‘5-ACCTTGTTACGACTT’) (100 mM, 1 µL each) (IDTDNA) and Power SYBR Green PCR 2x Master Mix (5 µL, Life Technologies Ltd.) to amplify a 123 bp fragment [26]. Amplification and quantitation consisted of 15 min of denaturation at 95 °C, 40 cycles of annealing at 95 °C for 15 s, 60 °C for 60 s and concluded with a holding temperature of 4 °C.

For the standard curve, serial dilutions of fresh mid-log phase *E. coli* MG1655 bacteria with known colony forming units (CFUs) were used. Calculated total cell counts are expressed as Gene Copy Numbers (GCNs).

### 2.5. Protein Gel Electrophoresis

To analyze the protein composition of *E. coli*, sodium dodecyl sulfate polyacrylamide gel electrophoresis (SDS-PAGE) was conducted. Protein contents were quantitated using the Bio-Rad Bradford Protein assay (Bio-Rad Laboratories Inc., Hercules, CA, USA) [27]. The sample volume needed for 10 µg protein per well was calculated based on the sample protein concentration. Sample solutions consisted of the calculated volume of the original samples, 2.5 µL of 4x sample buffer, 1 µL of 10x reducing agent Dithiothreitol (Sigma Aldrich, San Louis, MO, USA) and MQ-H_2_O for the difference to 10 µL total volume. Samples were heated to 70 °C for 10 min and allowed to cool to approximately 25 °C.

For running buffer, 20 mL NuPAGE MOPS 20x stock (Invitrogen, ThermoFisher Scientific, Waltham MA) was mixed with 380 mL of MQ-H_2_O and added into the assembled gel box. Samples were briefly centrifuged to collect the 10 µL volumes prior to loading on the gel.

The precast NuPAGE 4–12% Bis-Tris Gel from Invitrogen (ThermoFisher Scientific, Waltham, MA, USA) was loaded with a prestained molecular weight (MW) marker with known protein fragments (6.5, 14.4, 21.5, 31, 45, 66.2, 97.4, 116.2 and 200 kD, Bio-Rad Laboratories Inc., Hercules, CA, USA) and 10 µL of each protein sample at a constant voltage of 180 V for ~45 min. After 45 min, the gel was equilibrated in MQ-H_2_O and submerged in the Bio-Rad silver stain (Silver Stain Plus Kit #1610449) for 1 h [28]. The gel was destained in MQ-H_2_O on an orbital shaker for 8 h to reduce background and amplify the intensity of the protein bands.

### 2.6. Antibiotic Resistance

After collection, the samples were diluted 10x in Luria Bertani (LB) Broth (BD BBL™, Becton, Dickinson and Co., Franklin Lakes, NJ, USA) and incubated overnight at 37 °C. The next day, 100 µL of each sample was plated on Mueller-Hinton agar for the disc diffusion test. Sensi-Disc dispenser (BD BBL™ Sensi-Disc™, Becton, Dickinson and Co., Franklin Lakes, NJ, USA) was used to place eight antimicrobial susceptibility test paper discs on each plate, impregnated with commonly used antibiotics (30 µg of Tetracycline (TE-30), 10 µg of Ampicillin (AM-10), 30 µg of Cephalothin (CF-30), 10 µg of Gentamicin (GM-10), 10 µg of Imipenem (IPM-10), 5 µg of Ciprofloxacin (CIP-5), 23.75/1.25 µg of Sulfamethoxazole-Trimethoprim (SXT) and 75 µg of Cefoperazone (CFP-75).

### 2.7. Statistical Analysis

Statistical significance was calculated to compare stock samples and collected aerosol samples expressed resistance with or without 10% PBS at both 25 °C (room temperature, RT) and 4 °C. Statistical analysis was also completed by using various t-tests to determine statistical significance using RStudio (RStudio 2022.12.0 + 353, Posit, PBC). The null hypothesis stated that the mean percent development of resistance of stock samples would equal the mean development of resistance expression of the collected aerosol samples.

## 3. Results

**Figure 2:**
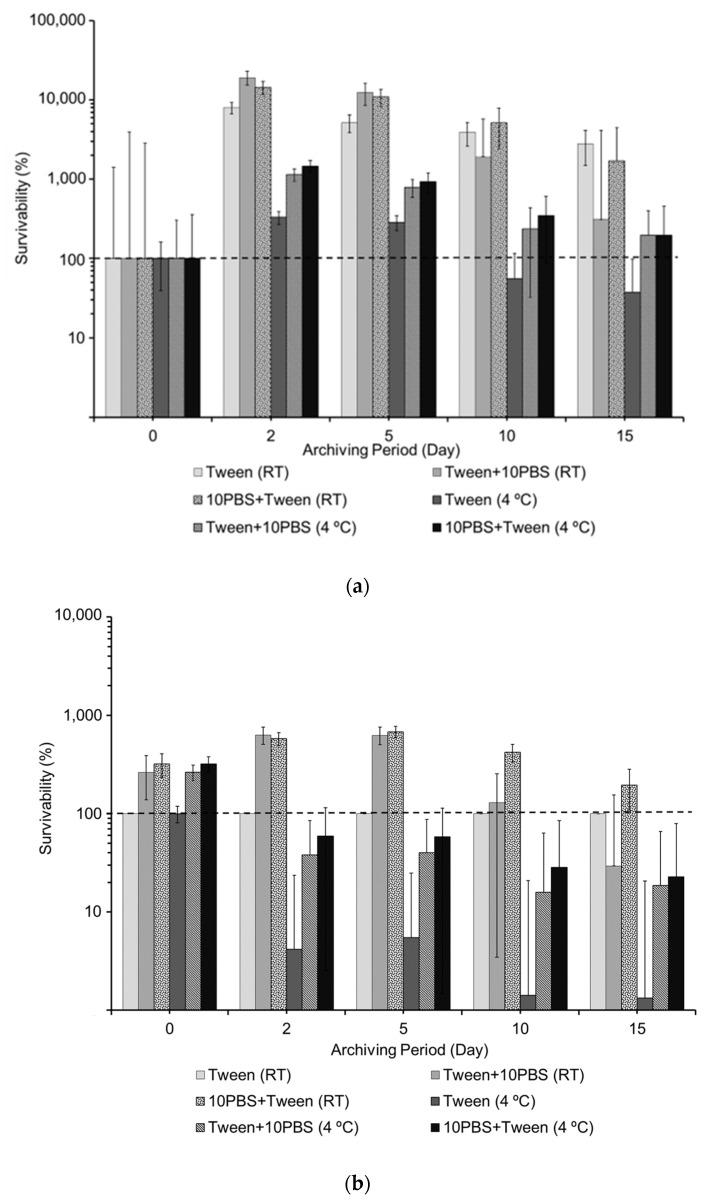
Survivability of the *E. coli* samples collected by the LCP-WWC at 55” H_2_O with the addition of PBS to 10% final concentration and archived at different temperatures for 10 days. (**a**). Values are compared to the CFU counts of the corresponding Day 0 samples as 100%. For example, Day 0 samples collected in Tween-20 and PBS were considered 100% and Day 2, 5, 20 and 15 samples collected in Tween-20 and PBS were compared to the same Day 0 sample (**b**). Values are compared to the CFU counts of each specific sample collected in Tween-20 and stored at RT as 100%.

The results suggest that the short-term exposure of the *E. coli* vegetative cells to extreme conditions (300 L/min airflow, 55” pressure drop, 98 m/s) causes a quiescent state in the collected bacteria, resulting in the dramatic, albeit transient, loss in culturability. However, based on the culturable and DNA-based total counts of the collected and archived aerosol samples, during the quiescent state, the viability of the bacteria, including the integrity of the cell membrane and the intactness of DNA, is maintained. The culturability of the cells was recovered within a two-day period, especially when stored at RT in the presence of PBS. Figure 2 shows that in the RT samples, percent culturability increased by 2 magnitudes by Day 2 and slowly decreased until Day 15. Culturability of samples archived at 4 °C increased 10-times when amended with PBS. While an increase in culturability was still detected in the samples that were not amended with PBS, those amended with PBS showed a 2-times greater increase compared to samples without PBS. There was a significant difference from Day 0 to Day 2 and Day 0 to Day 5 in samples archived at RT but not in samples archived at 4 °C. There was also a significant difference between Day 5 samples archived at RT and 4 °C. There was no significant difference between samples with 10% PBS added before or after the Tween solution.

**Figure 3:**
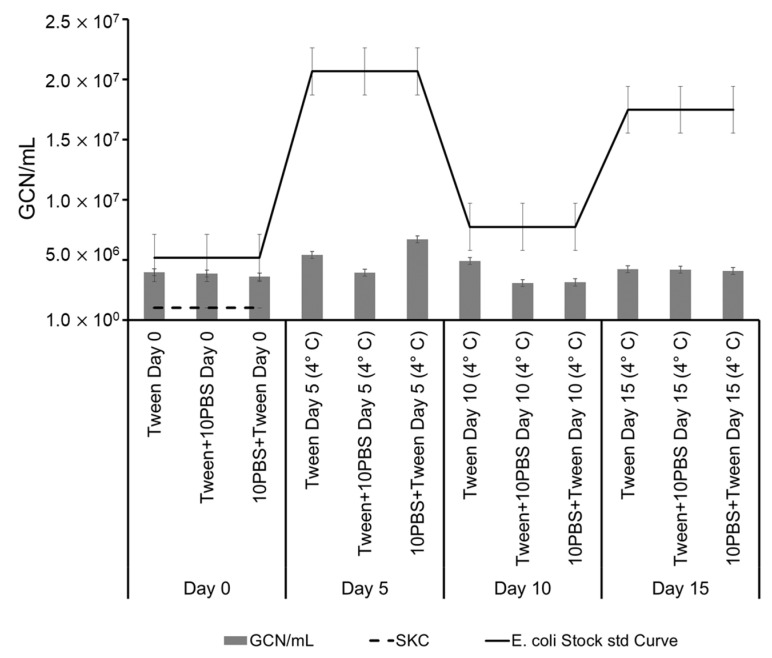
Real-time PCR analysis of the *E. coli* samples archived for 15 days at 4 °C collected in Tween, amended with 10% PBS (Tween + 10PBS) or being added to 10% PBS (10PBS + Tween). The GCN/mL values for the samples collected on Day 0 with the SKC impinger are indicated with a dashed line. The solid line represents the non-aerosolized *E. coli* stock.

**Figure 4:**
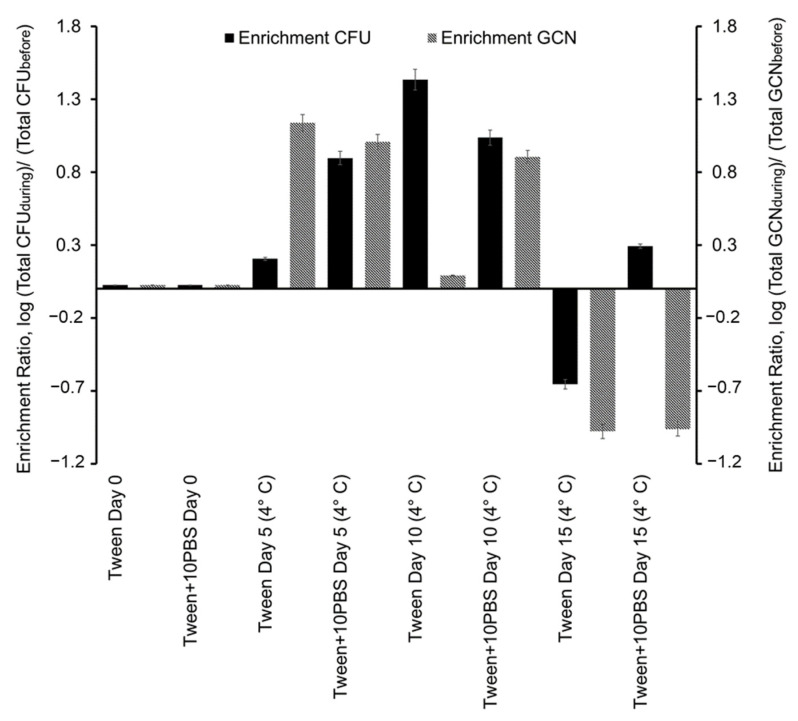
Enrichment ratio of the log of the airborne bacterial concentration based on viable colony forming units (CFUs), left axis, and on bacterial genome copy number (GCN), right axis, for the aerosol samples stored at 4 °C (during) compared to the total amount of CFUs or GCNs nebulized into the chamber (before). The baseline of 0 is in reference to the ratio of Day 0 to itself (Day 0).

However, after two days of archiving, the samples containing PBS exhibited a recovery in culturability, especially when stored at RT. There was no significant effect on culturability whether the samples were amended with 10% PBS during or after collection in Tween-20. Figure 3 shows the GCN/mL of aerosol samples in comparison with *E. coli* stock standard curve, in which, after Day 0, there is a magnitude of difference between GCN/mL. The difference showed when comparing the ratio of Total CFU on and after Day 0 and GCN on and after Day 0 (Figure 4). Ratios were the absolute values of the log of differences between the aerosol samples of the day and the Day 0 aerosol samples. The CFU increased after Day 0 with the largest ratio, increasing the values by 1.43-times from the Day 0 samples on Day 10 samples archived in Tween-20. The highest ratio increase for GCN was on Day 5 with samples archived in Tween-20, with samples increasing 1.13-times the Day 0 samples. There was also an increase for all samples compared to Day 0, except for Day 15 samples. Day 15 samples most likely decreased due to degradation of DNA and cell death as there was a decrease in culturability on Day 15.

Real-time PCR analysis showed that the as-collected samples contain higher amounts of DNA than expected based on their CFU counts (Figure 3 and Figure 4). However, adding PBS to the samples seemed to result in a recovery of culturability, especially in the samples that were archived at RT. As time progressed, the gap between CFU counts and DNA content stayed consistent, with GCN ratios being twice the amount of CFU ratios. The *E. coli* cells were likely still affected by the impact of sampling, resulting in a slowed metabolism after Day 0, which explains why fewer CFUs were recovered during archiving between Days 5 and 15. When bacteria are exposed to extreme environmental stressors, they can experience cell wall and membrane damage, causing a release of DNA [19,29]. The study of King and McFarland, 2012 showed that culturability and DNA integrity can be affected by mechanical stressors after aerosolization [19]. For cells to survive after experiencing damage, they must regulate repairs, including processes to restore DNA integrity, for example, via the SOS pathway of DNA repair in bacteria [30]. This likely explains the dramatic recovery of CFU and GCN values of the Day 5 and Day 10 samples as compared to Day 0 samples (Figure 4).

**Figure 5:**
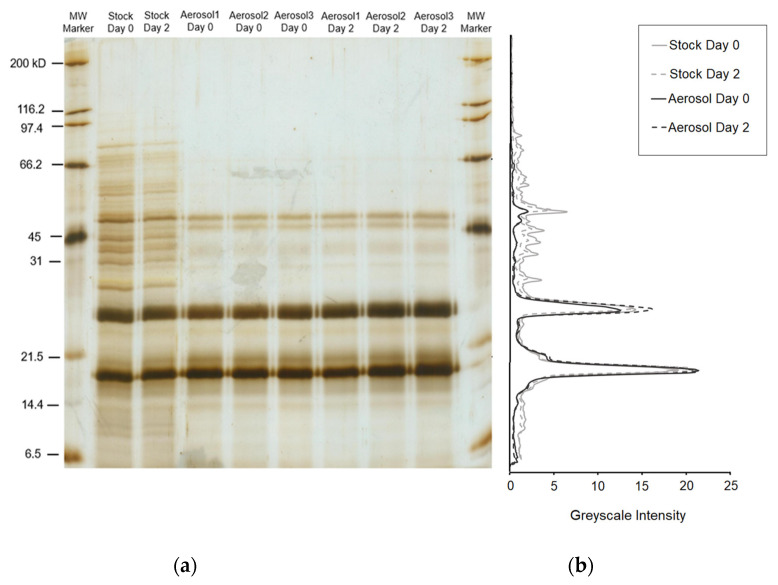
(**a**) Sodium dodecyl sulfate (SDS) gel electrophoresis results stained by the Silver Stain Plus kit (Bio-Rad) show continued protein synthesis of ribosomal proteins and a lower synthesis of non-essential proteins. (**b**) The greyscale intensity showing the peaks in bands of proteins for stock and aerosol samples, also indicating the kD values of the marker without including the peaks for easier visibility.

Figure 5a shows that the bands for most proteins are missing after aerosolization and collection while the essential (ribosomal) proteins are still present, indicating that the bacteria went into quiescence. Ribosomal proteins are any proteins working with rRNA for translation, enabling the cell to synthesize the essential proteins to survive while halting non-essential proteins, most likely to conserve energy [10]. Dark bands indicating proteins appear between 14.4–21.5 kD and 21.5–31. Ribosomal proteins in this region consist of S2–S7 proteins, and many of these proteins can be found in major ribosomal subunits, such as 30S, assisting in binding to 16S rRNA [31,32,33]. The intensity of the bands for ribosomal proteins is nearly 20-times higher than other faint bands, indicating a higher abundance of these proteins (Figure 5a).

**Figure 6:**
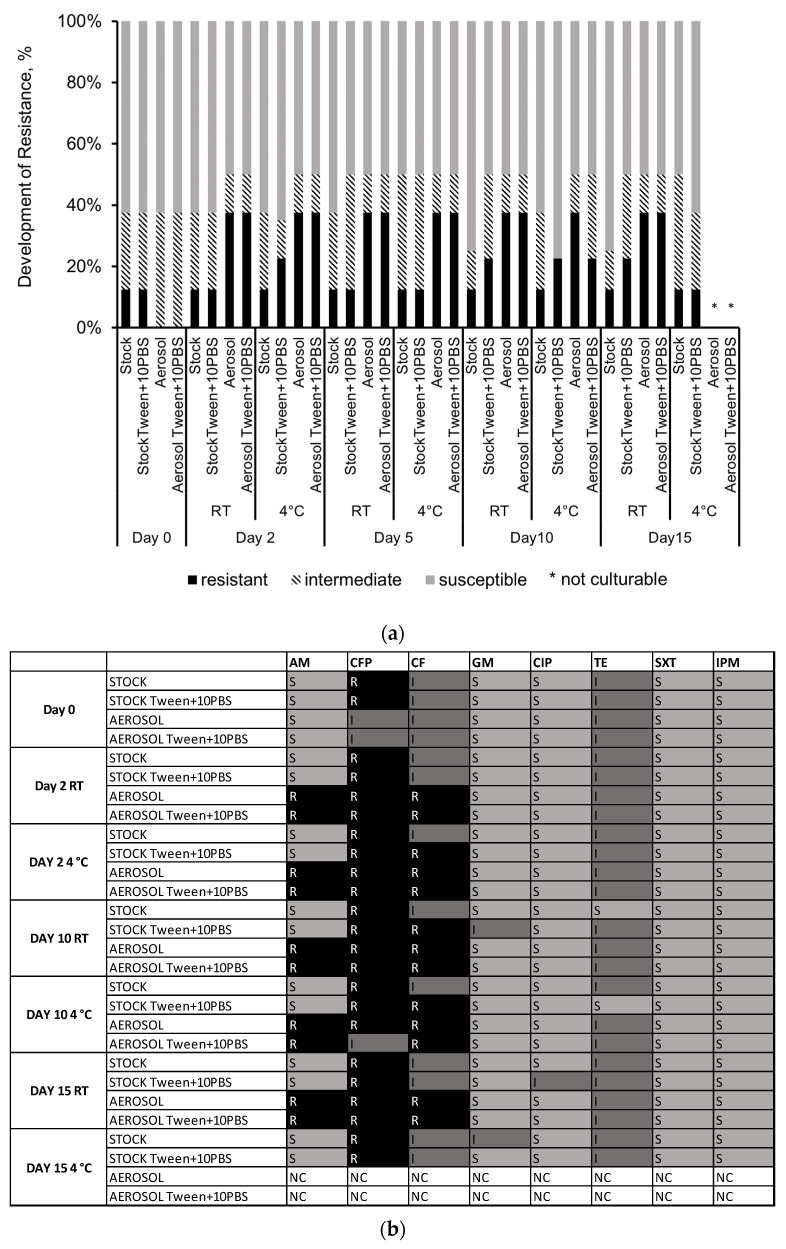
(**a**) The percentage of resistant, intermediate, and susceptible responses in each sample archived at either 4 °C or RT as collected with Tween-20 (Tween) or collected with Tween and amended with PBS (Tween + 10PBS) were recorded over a 15-day period. Samples that were not culturable are denoted by an asterisk. (**b**) Heat map showing Resistant (black), Intermediate (dark grey), Susceptible (light grey) or Not Culturable (white) responses to the Kirby–Bauer susceptibility test antibiotics (Ampicillin (AM), Cefoperazone (CFP), Cephalothin (CF), Gentamicin (GM), Ciprofloxacin (CIP), Tetracycline (TE), Sulfamethoxazole trimethoprim (SXT) and Imipenem (IPM)). Stock *E. coli* (S), nebulized liquid (N) and collected aerosol (A) sample.

The cell’s drive to maintain homeostasis may play a role in the development of antibiotic resistance by opening/gating the membrane channels to alleviate mechanical stress. Antibiotic susceptibility was tested to gather information on external environmental and mechanical stressors affecting antimicrobial resistance in the aerosol samples. Figure 6 shows that the collected *E. coli* cells expressed resistance mostly to ampicillin and cephalothin, both cell wall synthesis inhibitors. None of the control group or stock samples expressed resistance to ampicillin. Two samples showed intermediate susceptibility to cephalothin, while the stock samples on Day 0 expressed resistance to cefoperazone. Antibiotic resistance in the collected aerosols was not detected directly after collection on Day 0; however, during archiving, the bacteria expressed resistance, mainly, to cell wall synthesis inhibitors and maintained this resistance for the next 15 days, with some of the aerosols losing culturability by the 15th day. Intermediate susceptibility to Tetracycline, a DNA synthesis inhibitor, was also detected in the archived samples.

While there was a difference in culturability, no significant effect was detected on the development of resistance in the samples that were amended with 10% PBS. Figure 6 shows an increased resistance consistently for every aerosol sample compared to its relative stock sample. This percentage did not vary significantly from day to day, showing an average of 12.5% resistance in stock samples and 37.5% in aerosol samples, with an increase of 25% resistance in aerosols compared to stock samples which were not exposed to aerosolization stress. There was a decrease in resistance in aerosols on Day 10 archived at 4 °C and non-culturable bacteria in the aerosol samples by Day 15.

## 4. Discussion and Conclusions

Design modifications were made to an existing wetted wall cyclone to develop a Low Cutpoint Wetted Wall bioaerosol sampling cyclone with an aerosol sampling flow rate of 300 L/min and a continuous liquid outflow rate of about 0.15 mL/min at a pressure loss of 13.7 kPa (55 inches H_2_O). The cutpoint of the aerosol-to-hydrosol efficiency was 0.3 µm AD, with an average collection efficiency of 90% for single *E. coli* aerosols. The effect of aerosolization and the transient exposure to high air velocity of approximately 98 m/s during the collection of the bacteria resulted in a change in metabolism, culturability and antibiotic resistance. Cells entered a phase of quiescence or dormancy recovering by Day 2 with an increased culturability and expression of antibiotic resistance. This period of quiescence and decreased protein could be linked to cells using a period to recover from extreme stress and utilizing cell mechanics for DNA and protein repair. Previous research has shown that there was an increase in resistance to cell wall synthesis inhibitors and an increase in the activity of efflux pumps when bacteria were exposed to aerosolization stress. The increased turgor pressure cells are exposed to a change of environment, from an aqueous solution to aerosol, which could impact the integrity of the cell and, thus, the cell would need to compensate by utilizing membrane proteins, such as efflux pumps and mechanosensitive channels, to alleviate pressure on the cell [4]. These mechanisms have also been found to help bacteria evade antibiotics and could explain why the mechanical response also leads to antibiotic resistance [34,35]. Other mechanisms could also include osmoregulators, such as EnvZ or OmpR, to regulate porins after the downshock of changing osmotic environments [36]. It seemed to take approximately 2 days to recover from the initial stress of high-velocity aerosolization and then cells remained viable until Day 15. The implication of an increase in antibiotic resistance in bioaerosols maintained for nearly 15 days could increase the exposure and transmission of pathogens. Results indicate that the bacteria first expressed resistance to cell wall synthesis inhibitors, ampicillin and cephalothin, in all aerosol samples, with an increase in resistance in every sample compared to the non-aerosolized stock suspension, except for Day 15, when aerosol samples lost viability. Interestingly, stock samples that were amended with PBS showed an increase in resistance on Days 2, 10 and 15, most likely due to the increased support PBS offered the cells. After transient exposure to high air velocity, bacterial cells halted protein synthesis under stress and exhibited only essential, e.g., ribosomal, proteins during the first two days of collection and archival (Day 0 and Day 2). This further shows that the cells kept up essential functioning as they recovered from mechanical stress experienced and were then viable on Day 2, although many of the proteins were still not synthesized compared to the stock samples. Future work will aim to delineate genetic expression that could have impacted the quiescence, protein synthesis and antibiotic resistance responses of the *E. coli*. This study indicates that environmental changes and extreme environmental conditions cause transient and potentially permanent changes in aerosolized bacteria at the molecular level.

## Data Availability

The data presented in this study are available on request from the corresponding author.
